# Identification of *BnaYUCCA6* as a candidate gene for branch angle in *Brassica napus* by QTL-seq

**DOI:** 10.1038/srep38493

**Published:** 2016-12-06

**Authors:** Hui Wang, Hongtao Cheng, Wenxiang Wang, Jia Liu, Mengyu Hao, Desheng Mei, Rijin Zhou, Li Fu, Qiong Hu

**Affiliations:** 1 Oil Crops Research Institute of Chinese Academy of Agricultural Sciences/Key Laboratory for Biological Sciences and Genetic Improvement of Oil Crops, Ministry of Agriculture, No. 2 Xudong 2nd Road, Wuhan 430062, P.R. China

## Abstract

Oilseed rape (*Brassica napus* L.) is one of the most important oil crops in China as well as worldwide. Branch angle as a plant architecture component trait plays an important role for high density planting and yield performance. In this study, bulked segregant analysis (BSA) combined with next generation sequencing technology was used to fine map QTL for branch angle. A major QTL, designated as *branch angle 1 (ba1*) was identified on A06 and further validated by Indel marker-based classical QTL mapping in an F_2_ population. Eighty-two genes were identified in the *ba1* region. Among these genes, *BnaA0639380D* is a homolog of *AtYUCCA6*. Sequence comparison of *BnaA0639380D* from small- and big-branch angle oilseed rape lines identified six SNPs and four amino acid variation in the promoter and coding region, respectively. The expression level of *BnaA0639380D* is significantly higher in the small branch angle line Purler than in the big branch angle line Huyou19, suggesting that the genomic mutations may result in reduced activity of *BnaA0639380D* in Huyou19. Phytohormone determination showed that the IAA content in Purler was also obviously increased. Taken together, our results suggested *BnaA0639380D* is a possible candidate gene for branch angle in oilseed rape.

Rapeseed is one of the most important oil crops worldwide and yield enhancement is very important for achieving high production profit. As the arable lands gradually decrease due to growing population, high-density planting is a good approach for high efficiency of resource utilization^1^,^2^,^3^. Among all the agronomic traits under domestication process, plant architecture, e.g. leaf angle or branch angle, has played an important role in the adaption to high-density planting at present^4^. Genetic gain of yield has been enhanced in some crops, especially in maize, through breeding for high-density tolerance^2^,^3^. Therefore, optimized plant architecture suitable for high-density planting is regarded as one of the most important goals to improve the yield in oilseed rape, as well as in other cultivated crops.

Plant architecture is usually defined as the three-dimensional structure of aerial parts of plant, which is determined by plant height, branch pattern and number, leaf shape as well as morphology of other organs^5^,^6^,^7^. Branch angle is one of the most important traits of plant architecture, as it determines the optimum planting density and influences yield through affecting photosynthesis efficiency^8^. Smaller leaf inclination angle in sorghum caused solar radiation to penetrate deeper into the canopy and is predicted to increase the biomass yield^9^. Erect leaf trait can enhance photosynthetic efficiency and allow for increased planting density, thus leading to higher grain yield in maize^10^. Tiller angle in rice has attracted interests since the past decades because of agronomic importance^11^,^12^.

A multitude of results showed that tiller or leaf angle is a quantitative trait, which is mainly controlled by major genes and influenced by minor QTL. QTL/genes for tiller or leaf angle have been identified and investigated extensively. Some characterized genes shed lights on the mechanisms for the genetic control of leaf or tiller angle. In Arabidopsis, the auxin receptor mutant *tir1-1* has lateral branches that are less vertical^13^. *Indeterminate domain 15 (Idd15*)/*shoot gravitropism 5 (sgr5*) mutants displayed increased orientation angles in both branches and siliques^14^. *Tiller angle control1 (TAC1*) and *LAZY1* were two important genes modulating rice tiller angle^15^,^16^. *Loose Plant Architecture1 (LPA1*) regulates both tiller angle and leaf angle in rice by controlling the adaxial growth of tiller node and lamina joint^17^. Rice *PROG1* encoding a putative finger transcription factor with C-terminal EAT-like repression domain also regulates tiller angle^18^. *ZmTAC1* in maize, the orthologous of rice *TAC1*, has been demonstrated to be a major quantitative trait locus determining leaf angle^19^. Quite a few homologs of genes regulating branch angle in monocots also function in dicots. *AtLAZY1* in *Arabidopsis* has been demonstrated to regulate branch angle^20^. *TAC1* was identified as the candidate gene for branch angle in peach, a dicot tree species^21^.

Auxin or polar auxin transports (PAT) were proven to be involved in the gravitopism and thereby regulating tiller angle in rice. Increasing the level of a polar auxin transporter OsPIN2 resulted in enlarged tiller angle^22^. The amount of PAT was greatly enhanced in *lazy* mutant and thus the endogenous IAA distribution in shoot was changed, leading to increased tiller angle^15^,^23^. Recently, strigolactone (SL) biosynthetic or signaling mutants were identified to rescue the spreading phenotype of *lazy1*, suggesting that SLs can inhibit auxin biosynthesis and attenuate rice shoot gravitropism^14^. Potato plants overexpressing *AtYUCCA6* displayed smaller angle between petiole and stem and other high-auxin phenotypes, such as increased height, erect stature and longevity^24^. YUCCA has long been revealed to be involved in the tryptamine (TAM) pathway, which is one of the four tryptophan-dependent auxin synthesis pathways^25^. Recently, a two-step pathway of IAA biosynthesis in *Arabidopsis* from tryptophan was demonstrated^26^,^27^,^28^,^29^. YUCCAs encode flavin monooxygenases which convert indole-3-pyruvic acid (IPA) to IAA in the second step of the two-step pathway of IAA biosynthesis^25^. Overexpression of *YUCCA*s in *Arabidopsis* or rice resulted in high level of free IAA and therefore generated development defects associated with IAA accumulation^30^,^31^,^32^. *YUCCA* orthologous in other species also appeared to function in auxin synthesis based on their effects on plant development^32^,^33^,^34^. Similar to *AtYUCCA6*, other *YUCCA* orthologous involved in auxin synthesis may also play a role in the regulation of branch angle.

QTL mapping for genetic dissection of quantitative traits is useful for map-based cloning of related genes and marker-assist selection in plant breeding. However, the identification of polymorphic markers and genotyping of individuals from large segregation population is labor and cost consuming^35^. The rapid development of high throughput sequencing methods has helped quick identification of polymorphic markers and quantitative trait locus (QTL) by genotyping^35^. Bulked-segregant analysis (BSA) was widely used to rapidly identify markers linked to target genes or QTL by genotyping only two bulked DNA samples composed two opposite extremes of the interested trait in a segregating progeny^36^. Several QTL were rapidly identified by the combination of whole-genome re-sequencing with BSA, which was also referred as QTL-seq^37^,^38^,^39^,^40^,^41^. Recently, more QTL and candidate genes for agronomic traits were identified by QTL-seq, verifying the effectiveness of this approach^42^,^43^,^44^,^45^.

Compared with the remarkable progress in the above mentioned plant species, study on branch angle in *Brassica napus* was just on the threshold. Significant negative genetic correlations between branch angle and yield per plant were observed^46^. Twenty-five QTLs significantly associated with branch angle were identified after performing genome wide association study (GWAS) in a diversity panel^47^. Branch angle is as crucial for the establishment of ideal plant architecture and yield improvement in *B. napus* as in other crops. Branch angle was significantly decreased with the increasing of row spacing under higher plant densities^48^. Alteration of branch angle was considered to be well-suited for efficient light interception and easy operation of combine harvesting under higher plant density^48^. In this paper, we conducted whole genome sequencing of two DNA bulks from plants with extreme branch angle selected from an F_2_ population. Genome-wide SNP analysis allowed the detection of a genomic region harboring the major branch angle QTL and candidate genes were selected. Taken the advantage of conventional QTL analysis, expression pattern and phytohormones determination, we provided evidence that one gene which is the homolog of *AtYUCC6* encoding flavin monooxygenase, located at chromosome A06 is a candidate gene for branch angle in *B. napus*.

## Results

### Inheritance of branch angle in *B. napus*

**Figure 1:**
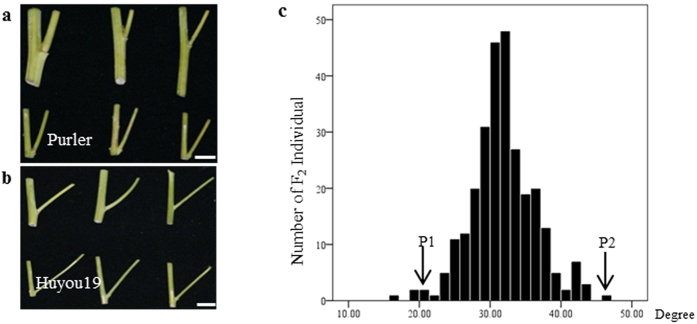
Phenotypes of Huyou19 and Purler with different branch angle and the frequency distribution of branch angle in F_2_ population. (**a**) Purler (P_1_); (**b**) Huyou 19 (P_2_); Upper row: Angle of the lowest branch, lower row: Angle of the top branch. Bar = 2 cm. (**c**) Frequency distribution of branch angle of 277 individuals in F_2_ population.

*B. napus* lines Huyou19 and Purler, harboring big and small branch angle respectively (Fig. 1a), were used to construct a segregation population. The average branch angle of Huyou19 (P_2_) was approximately 32 degrees larger than that of Purler (P_1_). The branch angle in F_2_ population including 277 randomly selected individuals displayed continuous variation, but not a complete normal distribution, showing a skewed distribution with a skewness of 0.086 (Fig. 1b). This result suggested that the branch angle trait in *B. napus* was quantitatively inherited. Genetic analysis by the mixed major gene plus polygene inheritance model using six generations (P_1_, P_2_, F_1_, F_2_, BCP_1_ and BCP_2_) showed that branch angle in *B. napus* was controlled by a pair of major gene with additive-dominant effects plus polygenes with additive-dominance-epistasis effects (unpublished data).

### Candidate QTL for branch angle identified by QTL-seq

**Figure 2:**
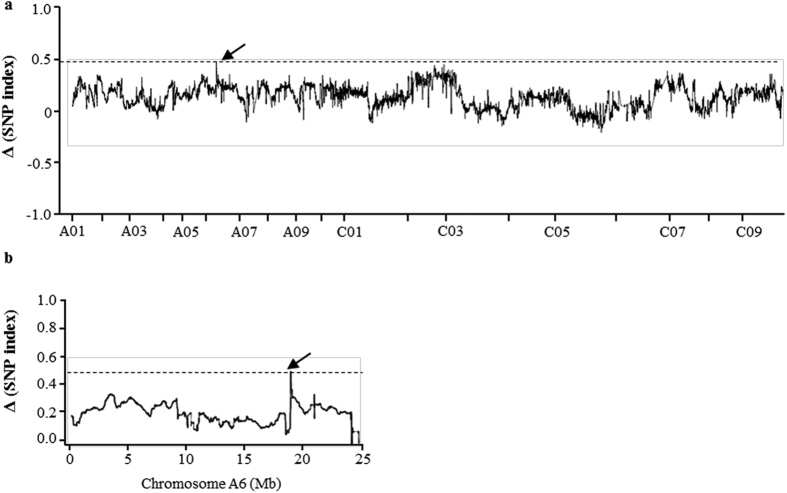
Δ SNP-index graphs from QTL-seq analysis. (**a**) X-axis represents the position of seven chromosomes and Y-axis represents the Δ SNP-index. SNP-index was calculated based on 1 Mb interval with a 5 kb sliding window. The Δ (SNP-index) graph was plotted with statistical confidence intervals under the null hypothesis of no QTL (*P *< 0.05). (**b**) One candidate QTL regions was identified in *B. napus* chromosome A06 (17.74–18.32 Mb interval) with the criteria that the SNP index in Big-pool was near 1, SNP-index in Small-pool was near 0 and the Δ (SNP-index) was above the confidence value (*P *< 0.05).

Genomic DNA of the two parents (Huyou19 and Purler) and the two pools (B-pool and S-pool) was sequenced by Illumina HiSeq 2500 sequencer and resulted in 10.45 million clean reads. Most of the reads obtained were high quality, with Q20 ≥ 93.57 and Q30 ≥ 88.25% (Table S1). Ultimately, 289,333,224 and 294,048,030 reads from the two DNA pools were generated (Table S2). In total, 2,171,317 SNPs were identified between two parents. Short reads of all the samples were aligned to the *B. napus* reference genome^49^. The SNP index of the two DNA pools was calculated for each identified SNP. SNP-index graph of B-pool and S-pool was shown in Supplementary Figure 1. Δ (SNP-index) was calculated and plotted to the genome position by combining the information of SNP-index in B-pool and S-pool (Fig. 2a). According to the null hypothesis, we chose peak regions above the threshold value (threshold value = 0.487) as the candidate region harboring major QTL for target trait (Fig. 2a). With 95% significance level, a genomic region (A06: 17.74–18.32 Mb) was found to hold the Δ (SNP-index) value above the threshold and was referred as the target region for branch angle (Fig. 2b).

### Validation of identified QTL by Indel markers

**Figure 3:**
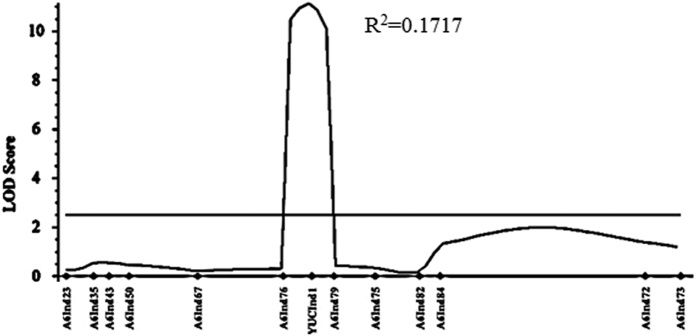
Genetic validation of branch angle QTL *ba1* in *Brassica napus* chromosome A06 using an F_2_ population. Linkage analysis confirmed the branch angle QTL with flanking markers A06Indel76 to A06Indel79. Candidate gene *BnaA06g39380D* was located at the peak of QTL interval. The location of *ba1* was generated using inclusive composite interval mapping (ICIM). The x-axis indicates the genetic distance of Indel markers. The y-axis indicates the LOD score of QTL. R^2^ is the phenotypic variance contributed by QTL *ba1*.

To verify the QTL predicted from QTL-seq, traditional QTL mapping was performed in the F_2_ population with 277 individual plants. In total, 658,715 Indel markers distributed at different genomic regions were detected according to high-throughput sequencing data (Table S3). Thirteen polymorphic markers located in the candidate region were used to genotyping 277 F_2_ plants and the data were used for linkage analysis. One major QTL located within about 82 kb physical distance on A06 was detected by Inclusive Composite Interval Mapping (ICIM) analysis for branch angle. This QTL with a LOD value of 11 accounts for 17.17% of phenotypic variance (Fig. 3). This result was consistent with that obtained from Δ(SNP-index) analysis of QTL-seq, supporting that a QTL locus for branch angle is located in the genome interval of A06, 17.74–18.32 Mb.

### Identification of candidate genes for branch angle

**Figure 4:**
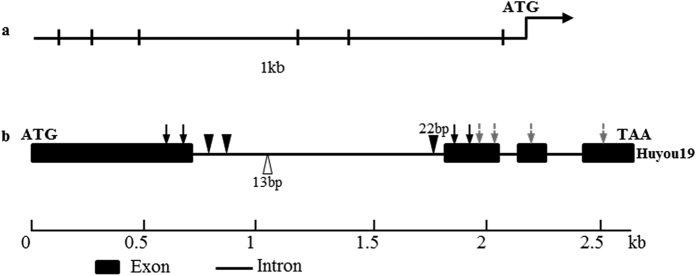
Structure and sequence variation of *BnaYUCCA6* between Huyou19 and Purler. (**a**) Comparison of promoter sequence of *BnaYUCCA6* between Huyou19 and Purler. Vertical bar represents nucleotide variation in promoter region; (**b**) Gene structure and mutation type of *BnaYUCCA6* in Huyou19 compared with Purler. Black and gray arrows represent non-synonymous and synonymous mutation in coding region, respectively. Inverted and positive triangles represent nucleotide insertion and deletion in the first intron, respectively. Size of exons and introns can be estimated using the scale bar at bottom.

Totally 82 genes were predicted in the A06: 17.74–18.32 Mb region delimited by two Indel markers A06Indel76 and A06Indel79. Three genes (*BnaA06g32200D*, *BnaA06g32210D* and *BnaA06g39380D*) were identified after filtration. *BnaA06g39380D* is a homolog of Arabidopsis *YUCCA6* which is involved in *de novo* auxin biosynthesis pathway^25^ and leaf angle modulation^24^. We nominated the *BnaA06g39380D* gene as “*BnaA.YUCCA6*.*a*” (Gene accession number: KX35886) based on the standard nomenclature of Østergaard and King^50^. To verify whether *BnaA06g39380D* is the candidate gene, genomic and complementary DNA (cDNA) sequences of this gene were cloned and sequenced from the two parents. Sequence comparison between the alleles of two parents showed that there are nine SNPs in the two exons both on DNA and cDNA levels (Fig. 4). Four amino acid variations were detected after aligning the predicted protein sequences of *BnaA06g39380D* in the two parent lines. Meanwhile, one 22-bp insertion in the first intron of Huyou19 allele was detected and a gene specific Indel marker (YUC6Indel1) was developed. This marker YUC6Indel1 was mapped right at the LOD curve peak for branch angle QTL (Fig. 3).

**Figure 5:**
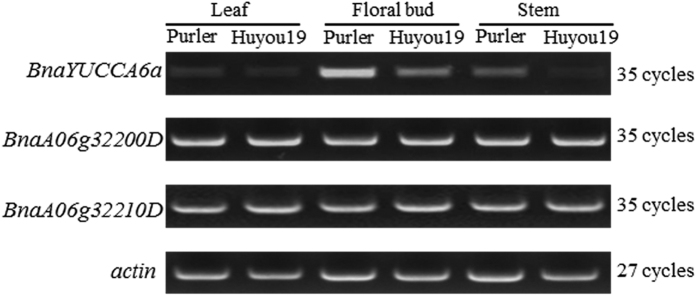
Transcript level of *BnaYUCCA6* in Huyou19 and Purler. The expression level of *BnaYUCCA6, BnaA06g32200D* and *BnaA06g32210D* was conducted by RT-PCR using gene specific primers. *Actin* was used as an internal reference gene with 27 cycles.

Expression patterns of YUCCA6 in two parental lines at different tissues were investigated to analyze whether the expression level of *YUCCA6* is associated with the variance of branch angle. The expression level of *YUCCA6* showed different among tissues. However, in all the three tissues examined, significantly higher *YUCCA6* expression was observed in the small branch angle line Purler than in the big branch angle line Huyou19 (Fig. 5). For other two identified genes, only one SNP variation has been detected in promoter region of gene *BnaA06g32200D*, homolog of which encodes hydrolases in Arabidopsis. We detected only one non-synonymous mutation in coding region of gene *BnaA06g32210D* which was predicted to encode a plant UBX-domain contained protein. None evidence showed that the function of these two genes is associated with plant hormone signaling or branch angle regulation. Meanwhile, no different expression of these two genes has been detected between two parents in three tissue samples (Fig. 5).

**Figure 6:**
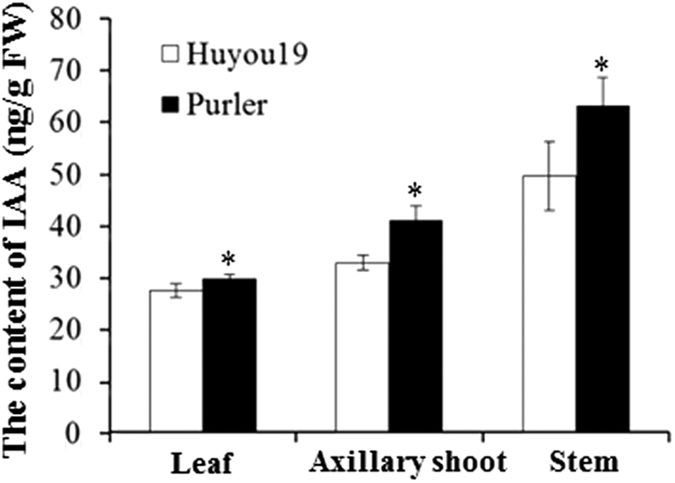
IAA of three tissue samples in Huyou19 and Purler. The data represent three independent experiments. Asterisks indicate a significant difference was detected between Huyou19 and Purler in the same tissue sample at *P < 0.05.

Previous results have emphasized the pivotal role of YUCCA in auxin biosynthesis. In order to investigate if IAA as a major auxin component is related to branch angle phenotype, IAA was measured in the two parental lines of different tissues. The result showed that IAA content was significantly increased in Purler compared with that in Huyou19 in leaf, axillary shoot and stem tissues (Fig. 6). All these results support that *YUCCA6* is a candidate gene for major QTL controlling branch angle in *B. napus*, though further genetic transformation assay needs to be performed for functional validation of the gene.

### Phylogenic analysis of *BnaYUCCA6*

**Figure 7:**
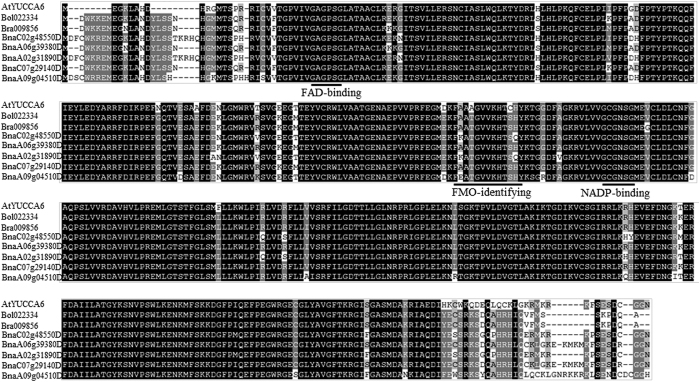
Comparison of amino acid sequences of AtYUC6 and YUC6-like genes from *Brassica napus*, *Brassica rapa* and *Brassica oleracea*. The sequences were aligned by ClustalW and edited by Gendoc. The conserved motifs of YUCCA proteins are shown at the appropriate location.

**Figure 8:**
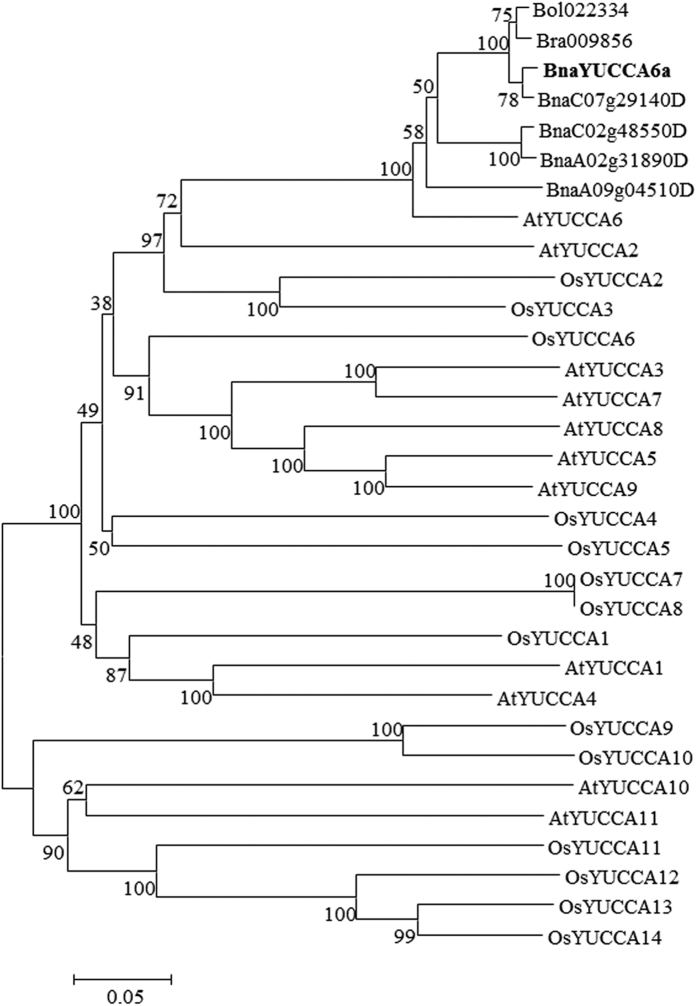
Phylogenetic analysis of YUCCA proteins. The YUCCA proteins from *Arabidopsis* (AtYUCCA), rice (OsYUCCA) and YUCCA6 protein in *B. napus* were aligned using ClustalW. The phylogenetic tree was constructed using the neighbor-joining algorithm with 1000 replication. Bar indicates 0.05 aa substitution per residue.

Eleven and fourteen *YUCCA* genes from *Arabidopsis* and rice, which are model plants for monocot and dicot species respectively, were extracted from the public database (http://www.dtd.nlm.nih.gov/). Five homologs were identified in the *B. napus* genome by using AtYUCCA6 as query against the “pseudomolecules” representative of the *B. napus* genome (version 4)^49^. Two AtYUCCA6 homologs were obtained from *Brassica rapa* and *Brassica oleracea* genome respectively. Alignment of amino acid sequences of YUC6-like genes from *B. napus*, *B. rapa* and *B. oleracea* showed that the motifs of YUCCA6 protein were highly conserved, all containing FAD-binding domain, FMO-identifying domain and NADP-binding domain (Fig. 7). A total of 32 YUCCA proteins were used for the construction of an unrooted phylogenetic tree. Phylogenetic analysis showed that the homologs of AtYUCCA6 from Brassica species were clustered in one individual group, indicating the conservation of YUCCA proteins among Brassica species during evolutionary process. Apart from very high similarity to the counterparts from *B. napus*, *YUCCA6* also showed in general a closer relationship with its orthologs in *B. rapa* and *B. oleracea* (Fig. 8). *YUCCA6* proteins from dicots *B. napus, B. rapa*, *B. oleracea* and *Arabidopsis thaliana* were clustered in a big group, which also implies their functional consistence.

## Discussion

Methods based on molecular marker development have made tremendous progress in genotyping and QTL mapping. For genetic map construction and genetic dissection of quantitative traits, QTL mapping using traditional markers is considered to be most reliable. However, traditional QTL mapping is usually conducted by genotyping a large number of individuals in a primary population and then fine mapping candidate genes in advanced populations, which is very time-consuming and painstaking^35^. QTL-seq integrated the advantages of BSA and high-throughput whole genome re-sequencing, has been proven to be an efficient and quick method to ascertain the genomic regions harbor QTL for quantitative traits. Previous results showed that QTL-seq can be applied in RIL or F_2_ populations to identify QTL for different agronomic traits undergone artificial or natural selective sweeps^37^. Since the first article published in yeast^36^, many QTL or candidate genes have been identified in different plant species by QTL-seq^38^,^39^,^40^,^41^,^42^,^43^,^44^. Previous results by genome wide association study (GWAS) showed that there are twenty-five QTL associated with branch angle in a diversity panel^46^. In this study, we identified one QTL for branch angle in *B. napus* by QTL-seq using an F_2_ population. This QTL was verified by traditional mapping approach and a candidate gene responsible for branch angle was predicted. The whole procedure took only 2.5 years including 1.5 years for F_2_ population construction. Although high-throughput sequencing is expensive now, it is still worthwhile since a lot of time and labor input can be significantly reduced. For a plant species which has a reference whole genome sequence such as oilseed rape, QTL-seq is a highly effective approach for identify major QTL or candidate genes for quantitative traits.

QTL-seq has rarely been applied in *Brassica* species^51^. Seventy SNPs associated with pod shatter resistance was identified by using similar approach. In their study, a major QTL *psr1* for pod shatter resistance was mapped by sequencing reduced representation libraries of two bulked samples of plants with contrasting pod shatter resistance from an F_2_ population using restriction digests and size selection^51^. This QTL was confirmed by traditional mapping with the F_2_ population and genome-wide association studies with diverse *B. napus* germplasm^52^. However, none candidate gene was predicted even though the QTL was confined within a 396 kb genomic region on A09 chromosome of *B. rapa* at that time. After two years, *SHP1* was predicted to be a candidate gene of the A09 locus using the whole genome sequence of *B. rapa* by another group^52^. Limiting factors for the failure of gene prediction in the previous study^51^ include lacking of *B. napus* reference genome sequence and re-sequencing information of parental lines at that time. Since there are usually frequent rearrangements of genome fragments between the A genomes from *B. napus* and *B. rapa*^53^,^54^, as well as among genomes from different varieties among *B. napus* lines^55^,^56^, genomic sequence information of parental lines is of great importance for candidate gene prediction within the targeted QTL region. Our study taking the advantage of re-sequencing the parental lines, made prediction of candidate genes easier by sequence comparison of genes within the 580 kb QTL genomic region.

As a quantitative trait, branch angle is certainly not only controlled by one locus. More loci could be detected when the threshold value of Δ (SNP-index) is decreased. In order to include as many loci as possible, 30 individuals with extreme phenotype from F_2_ population were selected to combine the DNA pool, which contained plants with relatively wide range of branch angle values. Higher percentage of F_2_ individuals included in the extreme bulks will increase the power to detect causal QTL despite allele frequency difference can be relatively smaller between bulks^40^,^57^. Nevertheless, the locus detected in this study with the highest Δ (SNP-index) value has the highest chance to be actually associated with the branch angle trait.

*YUCCA6*, an *AtYUCCA*6 homologous gene located at the A06 *ba1* QTL region was identified as a candidate gene for branch angle based on gene sequence variation, physical mapping, functional prediction by phylogenetic analysis as well as differential expression. YUCCA genes encode flavin monooxygenases and function in the second step in *de novo* auxin biosynthesis pathway^22^. Seedlings of the *yuc1yuc2yuc4yuc6* mutant accumulate more IPA, suggesting the YUCCA functioned in the step of IPA to IAA conversion^26^,^29^. Plants overexpressed *Arabidopsis AtYUCCA6* displayed high-auxin phenotypes in *Arabidopsis*, potato and tobacco, including reduced angle between petiole and stem^24^. These results suggest that AtYUCCA6 was involved in auxin biosynthesis modulating leaf angel as well as other auxin-related phenotypes. YUCCA proteins contain two conserved motifs for FAD and NADPH binding respectively. Mutations of the two binding sites completely abolished YUCCA function in *Arabidopsis*^58^. Sequence alignment showed that no amino acid variation was detected at the conserved motifs of YUCCA6a in the two parent lines, suggesting that the function of *YUCCA6* alleles in the two parent lines may be no different. However, six SNP variations were detected after comparison of promoter sequences of *YUCCA6* in the two parent lines. One SNP occurred in the cis-element bound by ARR1 (Arabidopsis response regulator) which functions as cytokinin receptor genes. Complex cross-talk between auxin and cytokinin and integration of hormone signaling are required for differentiation and maintenance of plant meristems as well as other developmental process^59^. Previous results showed that ARR1 activated the gene SHY2/IAA3 (SHY2) which functions as a repressor of auxin signaling and negatively regulates polar auxin transport^60^. Therefore, mutation within the cis-element of Purler promoter may result in loss of the suppression activity by cytokinin response regulator ARR1. Expression of *YUCCA6* may be influenced due to these SNPs in the promoter region and thus impact the auxin *de novo* synthesis, giving rise to the higher expression of *YUCCA6* and IAA content in Purler, leading to increased branch angle ultimately. All these analysis enables us to postulate that *YUCCA6* is the candidate gene for branch angle regulation. Studies with transgenic plants overexpressing *YUCCA6* in small branch angle lines will be needed for functional validation of this gene. Further study focus on the molecular characterization of *YUCCA6* may not only provide genetic resource to improve the yield of *B. napus* but also shed light on the role of auxin biosynthesis in branch angle regulation.

## Methods

### Plant materials and phenotyping for branch angle

One method for precisely measuring branch angle in *B. napus* was established previously^46^. At adult stage, the first and the last branch node of each plant was cut and packed in one envelop to dry. Picture of the branch node was taken by a digital camera (DSLR-A350, SONY, Japan) on a flat platform and analysed using AutoCAD software. The branch angle value of a plant was calculated as the average of branch angles from the first and the last branch node. Purler and Huyou19 which harbor small and big branch angle, respectively, were selected as the parental lines to develop F_2_ segregation population. A cross was made between Purler (female parent, P_1_) and Huyou19 (pollen donor, P_2_) to create F_1_ plants. F_2_ population was generated by self- pollination of F_1_ plants. All plant materials were grown in the field of Oil Crops Research Institute of the Chinese Academy of Agricultural Sciences (OCRI-CAAS), Wuhan, China. Equal amount of DNA from thirty of 277 F_2_ plants with the smallest branch angle (19.26–27.15 degrees) was mixed to form the small branch angle bulk (S-pool), and that from another thirty plants with the biggest branch angle (42.77–57.88 degrees) was mixed to form the big branch angle bulk (B-pool).

### Generation and analysis of NGS data

Sequence data of two bulks and two parents were generated by Illumina HiSeq 2500 and analyzed by Novogene (No. 38, Xueqing road, Haidian district, Beijing, http://www.novogene.com). In order to ensure the reads being reliable without artificial bias, such as low quality paired reads, which mainly resulted from base-calling duplicates and adapter contamination, raw data (raw reads) were firstly processed through a series of quality control (QC) procedures. BWA (Burrows-Wheeler Aligner) was used to align the clean reads from two DNA bulks against the reference genome of *B. napus*^50^,^61^,^62^. Alignment files were converted to BAM files using SAMtools software^63^. In addition, potential PCR duplications were removed using SAM tools command “rmdup”. If multiple read pairs have identical mapping site, only the pair with the highest mapping quality was retained.

SNPs or Indels were detected using the VariantFiltration parameter and annotated according to GFF3 files aligned with the reference genome by an efficient software tool ANNOVAR^64^. Homozygous SNPs between the two parents were extracted from vcf files. The genotype of one parent was used as the reference to calculate the number of reads of this parent’s genotype in the individuals of the offspring pools. The ratio of reads harboring SNP which is different from the reference sequence was calculated as the SNP index of the base site. Positions with SNP-index less than 0.3 were filtered out, as which may due to sequencing or alignment errors. Sliding window methods were used to present SNP index of the whole genome. The SNP index for each window was calculated as the average of all SNP index in that area of the genome. As usual, we defined window size as 1 Mb and step size as 10Kb according to the default setting. The difference of SNP index of the two pools was calculated as Δ (SNP index). Computer simulation was carried out to generate the confidence intervals of the SNP-index value under null hypothesis with no QTL, and the confidence intervals of Δ (SNP-index) were defined to be 95% as described previously^40^.

### QTL analysis by Indel marker

The QTL identified from QTL-seq were confirmed by conventional genetic linkage analysis. Reads from both parents were aligned to the *B. napus* reference genome (http://www.genoscope.cns.fr/brassicanapus/data) with BWA/SAMtools software. Gene specific Indel markers were exploited by comparison of candidate gene sequences between the two parents and then primers were designed by Primer Premier 5 based on sequence differences. PCR reaction was conducted using the following program: 5 min at 95 °C, 14 cycles of 30 s at 95 °C, 40 s at 64 °C and 1 min at 72 °C, then 24 cycles of 30 s at 95 °C, 40 s at 50 °C and 1 min at 72 °C. Individuals of the F_2_ population were genotyped using Indel markers exhibited clearly polymorphic between parental lines within the candidate region. Linkage analysis and QTL mapping was operated with the inclusive composite interval mapping (ICIM) procedure^65^. The ICIM of QTL was done by using QTL IciMapping software (http://www.isbreeding.net) and BIP (QTL mapping in bi-parent population) functionality. ICIM-ADD method was used to map. The largest *P* value for entering variables in stepwise regression of phenotype on marker variables was 0.001. The step size was 1 cM. A LOD score threshold of 2.5 was used to declare the existence of a QTL.

### Candidate gene analysis

Based on the sequencing data of parental lines, sequences of the predicted genes within the A06 17.74–18.32 Mb QTL region were compared between the parents. SNP sites with significantly different SNP-index (with SNP-index ≥ 0.8 in B-pool and ≤0.2 in S-pool) were selected as polymorphic marker loci. According to the annotation result of ANNOVAR, genes with SNPs causing stop gain or loss and non-synonymous mutation in their corresponding alleles were selected as candidate genes. Meanwhile, genes with SNPs in the promoter region prior to start codon ATG (≤1 kb) in their corresponding alleles were also selected as candidate genes.

### Expression analysis by semi-quantitative RT-PCR

We investigated the expression pattern of the candidate genes. Samples of leaf, stem and floral bud were collected from five different individuals of Purler and Huyou19 at bolting stage and pooled with equal amount of tissue from each plant. Total RNAs for all the samples were extracted with Trizol Reagent (Invitrogen, America). Reverse transcription was performed according to the instruction of FastQuant RT Kit (Tiangen, China). RT-PCR was performed as described previously using the primers listed in Table S5. The expression level of actin gene in *B. napus* was used to standardize the RNA sample for each RT-PCR. The reaction was conducted using following program: 5 min at 95 °C, 35 cycles of 30 s at 95 °C, 40 s at 56 °C and 1 min at 72 °C.

### IAA Quantification

Three tissue samples: leaf, stem and axillary shoot were used for IAA quantification. Samples were prepared and mixtures of compounds were separated by HPLC (Agilent 1200) and analyzed using a hybrid triple quadrupole/linear ion trap mass spectrometer (ABI 4000 Q‐Trap, Applied Biosystems, Foster City, CA, USA). IAA was quantified according to the method described previously^67^.

### Phylogenetic analysis of *YUCCA* genes

Blastp was performed against *B. napus* genome data using AtYUCCA6 protein sequence as query. YUCCA family members from *Arabidopsis* were downloaded from TAIR (http://www.Arabidopsis.org/). Rice *YUCCA* genes were downloaded from rice genome project (http://rice.plantbiology.msu.edu/). Multiple sequence alignment of YUCCA protein sequence from *Oryza sativa*, *Arabidopsis thaliana* and *B. napus* was performed using ClustalX2.0 with default parameters. Sequence alignment was further edited by Gendoc software. Phylogenetic trees were constructed by MEGA6.0 software using the neighbor-joining (NJ) method with 1000 bootstrap replications.

## Additional Information

**How to cite this article**: Wang, H. *et al*. Identification of *BnaYUCCA6* as a candidate gene for branch angle in *Brassica napus* by QTL-seq. *Sci. Rep.* **6**, 38493; doi: 10.1038/srep38493 (2016).

**Publisher's note:** Springer Nature remains neutral with regard to jurisdictional claims in published maps and institutional affiliations.

## Supplementary Material

Supplementary Dataset 1
