# Supplementary material for: Identification of BnaYUCCA6 as a candidate gene for branch angle in Brassica napus by QTL-seq
Source: Sci Rep. 2016 Dec 6;6:38493. doi: 10.1038/srep38493 (PMC5138835; doi:10.1038/srep38493)
Supplement: Supplementary Dataset 1 [file srep38493-s1.doc]

**Identification of a branch angle QTL located near *YUCCA6* in *Brassica napus* by QTL‑seq**

Hui Wang^1^, Hongtao Cheng^1^, Wenxiang Wang, Jia Liu, Mengyu Hao, Desheng Mei, Rijin Zhou, Li Fu, Qiong Hu*

Address: Oil Crops Research Institute of Chinese Academy of Agricultural Sciences / Key Laboratory for Biological Sciences and Genetic Improvement of Oil Crops, Ministry of Agriculture, No.2 Xudong 2nd Road, Wuhan 430062, P. R. China

*Corresponding author

^1^ These author contributed equally to the work.

**Table S1** Summary of sequencing date and quality

| Sample | Raw Base(bp) | Clean Base(bp) | Effective Rate(%) | Error Rate(%) | Q20(%) | Q30(%) | GC Content(%) |
| --- | --- | --- | --- | --- | --- | --- | --- |
| Huyou19 | 15,925,061,000 | 15,816,458,750 | 99.32 | 0.04 | 94.87 | 90.27 | 39.88 |
| Purler | 13,446,801,750 | 13,350,046,750 | 99.28 | 0.04 | 94.21 | 89.41 | 39.12 |
| Big-pool | 36,570,340,750 | 36,166,653,000 | 98.89 | 0.04 | 93.57 | 88.25 | 40.55 |
| Small-pool | 37,150,012,750 | 36,756,003,750 | 98.97 | 0.04 | 94.15 | 89.24 | 38.43 |

**Table S2** Sequencing depth and coverage statistics

| Sample | Mapped reads | Total reads | Mapping Rate(%) | Average depth(×) | Coverage at  least 1 (%) | Coverage at  least 4×(%) |
| --- | --- | --- | --- | --- | --- | --- |
| Huyou19 | 124,648,611 | 126,531,670 | 98.51 | 19.81 | 91.11 | 81.15 |
| Purler | 105,143,311 | 106,800,374 | 98.45 | 16.74 | 92.35 | 82.63 |
| Big-pool | 284,500,964 | 289,333,224 | 98.33 | 40.86 | 95.75 | 90.39 |
| Small-pool | 289,107,772 | 294,048,030 | 98.32 | 41.23 | 96.68 | 93.58 |

**Table S3** Indel detection and annotation

| Upstream | Exonic | Intronic | Splicing | Downstream | Upstream | Intergenic | Total |
| --- | --- | --- | --- | --- | --- | --- | --- |
| 113,027 | 21,295 | 145,864 | 1,717 | 87,093 | 29,172 | 260,547 | 658,715 |

**Table S4** Indel primers used for QTL analysis

| **Marker** | **Nucleotide variation in Huyou19** | **Nucleotide variation in Purler** | **Forward primer sequence** | **Reverse primer sequence** | **Length** |
| --- | --- | --- | --- | --- | --- |
| A06Ind23 | AATCAGACTGCTTTTT | A | TGAGATCTGTGAACTTGCTG | GGGTGATTAAACATCAAAACA | 139 |
| A06Ind35 | A | ACGACAATGTCTCG | ATGGCTACGTGTAAGCAAAT | AAAAATCAGAACCATTTGGA | 121 |
| A06Ind43 | A | AAGAAGATAGTTCCATGGC | TCCCTCATCTAGACAACCTG | TATGAGAAGCCGATGAATCT | 143 |
| A06Ind50 | GTATTTTTTTTTCTTTTATAAGCTTTCC | G | GATCTCATGGACCAAAGAAA | GATTAGGTGACAAAGGGTCA | 145 |
| A06Ind67 | GTGGAATATCTTTCAACC | G | TCTGAGAAACATTGAGGACC | GACAGATCCGCATAGTCAAT | 129 |
| A06Ind71 | GATGTAAAATCT | G | TCCGCAGACACCTATTGTAA | ATCCTTTCCACCGAGACAAT | 105 |
| A06Ind72 | CTCTCCCTCCCTCCCTG | C | GATCCGGTTCATCAAGTCTG | TGATTATCAGTCGAGAGCCA | 108 |
| A06Ind75 | T | TCTCCCTCCCTCC | ACCACTAATCCCATTCTCCT | CTGCTCATTTTTGTCGATCT | 108 |
| A06Ind76 | TTTTTATGCTCTTTTTA | T | TCAATGCCTCTCTCTTGTCT | ATTCGCTGAGAACAAATCAT | 153 |
| A06Ind79 | AACATCCATGATGT | A | ACCAGGCTGTAAATAAGCAG | TGGTGTTTTTAGTGCTCTTTC | 102 |
| A06Ind82 | CAATTATGCATATATATATATAT | C | TCAGATCAAACAAACACAAA | TTGTCTTTCGTAACTACTTTCA | 129 |
| A06Ind84 | GAGGAGGAGAATAAATAAA | G | GAGCTCTGAAAGATCGAAGA | TGCAGGTATAAGAAGAAGGC | 151 |
| YUCIndel | TCTCTAAACATTTAAAATATAG |  | TACCTAATTTCTGTAATTCGG | TGAGTAACAACATGGACAGC | 120 |

**Table S5** Primers used for gene structure and expression analysis

| **Primer** | **Forward primer sequence** | **Reverse primer sequence** | **Products** | **Use** |
| --- | --- | --- | --- | --- |
| YUCF2/R2 | CTAGCAAAAGCGAATTATGTA | AGATCTCACTCTTGTATGTTC | 2.8kb | Amplifying genomic DNA of YUCCA6 |
| YUCF6/R6 | GCCCCTCCTATATATATTGCAG | GCTACCCGTCTTCAAGTTAT | 1.4kb | Amplifying cDNA of *YUCCA6* for gene structure |
| YUCF7/R7 | CTTGTCTCAAAAAGAAAGGA | AATCAAGAGGAAACGGTCAA | 0.6kb | Amplifying cDNA of *YUCCA6* for semi-quantitative RT-PCR |
| A06g32200F/R | TGCCTATAACACTGTCACTG | TCAAAAGAGGGATTGCAGCT | 0.54kb | Amplifying cDNA of *BnaA06g32200D* for semi-quantitative RT-PCR |
| A06g32210F/R | TCATCTCAGGAGGTTGGTG | CGAAGGTAAACATCCTCTGT | 0.62kb | Amplifying cDNA of *BnaA06g32210D* for semi-quantitative RT-PCR |
| ActinF/R | TCTGGCATCACACTTTCTACAACGAGC | CAGGGAACATGGTCGAACCACC | 0.75kb | Used as internal control for semi-quantitative RT-PCR |


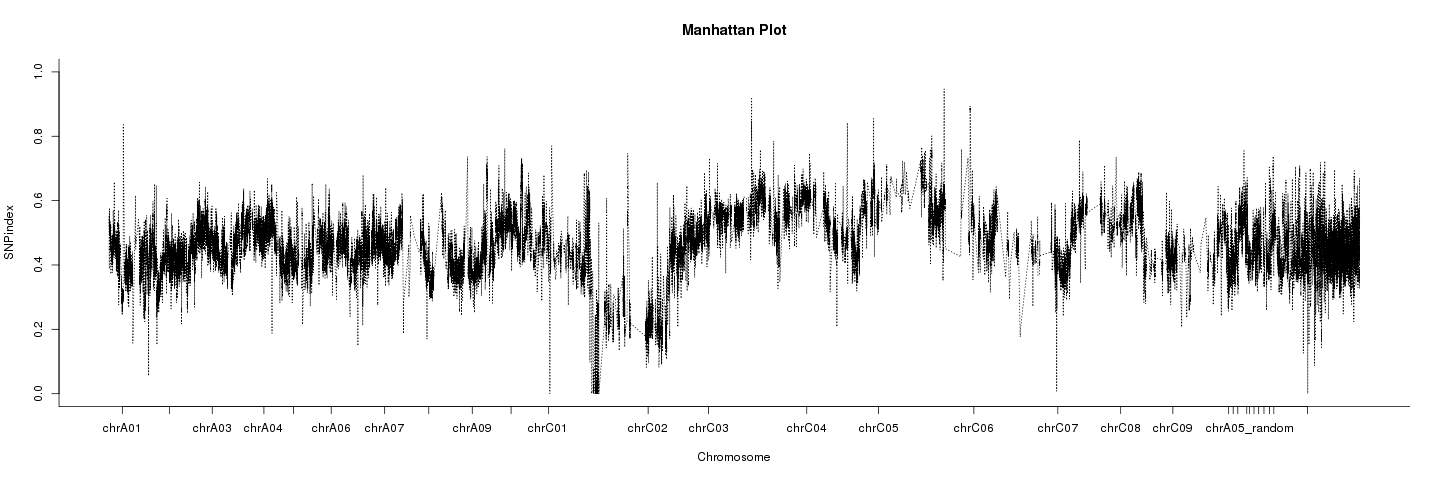


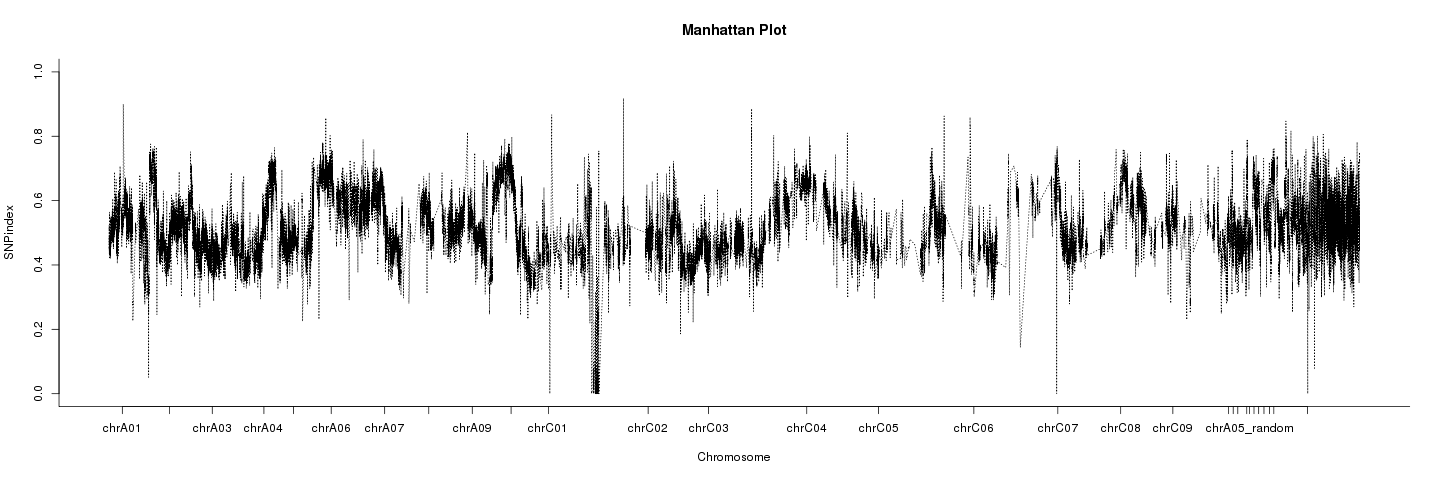


Figure S1 SNP-index of B-pool and A-pool. Figu
